# Effectiveness of Rotigotine plus intensive and goal-based rehabilitation versus Rotigotine alone in “de-novo” Parkinsonian subjects: a randomized controlled trial with 18-month follow-up

**DOI:** 10.1007/s00415-018-8792-0

**Published:** 2018-02-13

**Authors:** D. Ferrazzoli, P. Ortelli, G. Riboldazzi, R. Maestri, G. Frazzitta

**Affiliations:** 1 Department of Parkinson’s Disease, Movement Disorders and Brain Injury Rehabilitation, “Moriggia-Pelascini” Hospital-Via Pelascini 3, Gravedona ed Uniti (CO), 22015 Como, Italy; 2 Parkinson’s Disease and Movement Disorders Center, ASST dei Sette Laghi, Varese, Italy; 3 Parkinson’s disease Rehabilitation Center, Fondazione Borghi, Brebbia, Varese, Italy; 4 Department of Biomedical Engineering, Istituti Clinici Scientifici Maugeri Spa Società Benefit, IRCCS, Via per Montescano 3, Montescano, 27040 Pavia, Italy

**Keywords:** Parkinson’s disease, Rehabilitation, Multidisciplinary care, Dopamine agonists, DRT-related side effects

## Abstract

**Background:**

Dopamine Replacement Therapy (DRT) represents the most effective treatment for Parkinson’s disease (PD). Nevertheless, several symptoms are unresponsive to treatment and its long-term use leads to serious side effects. To optimize the pharmacological management of PD, dopamine-agonists are often prescribed to “de-novo” patients. Moreover, several studies have shown the effectiveness and the synergic effect of rehabilitation in treating PD.

**Objective:**

To evaluate the synergism between DRT and rehabilitation in treating PD, by investigating the short and the long-term effectiveness of a multidisciplinary, intensive and goal-based rehabilitation treatment (MIRT) in a group of patients treated with Rotigotine.

**Materials and methods:**

In this multicenter, single blinded, parallel-group, 1:1 allocation ratio, randomized, non-inferiority trial, 36 “de-novo” PD patients were evaluated along 18 months: 17 were treated with Rotigotine plus MIRT; 19 were treated with Rotigotine alone (R). The primary outcome measure was the total score of Unified Parkinson’s Disease Rating Scale (UPDRS). The secondary outcomes included the UPDRS sub-sections II and III (UPDRS II-III), the 6-Minute Walk Test (6MWT), the Timed Up and Go Test (TUG) and the amount of Rotigotine. Patients were evaluated at baseline (T0), 6 months (T1), 1 year (T2), and at 18 months (T3).

**Results:**

No differences in UPDRS scores in the two groups (total score, III part and II part, *p* = 0.48, *p* = 0.90 and *p* = 0.40, respectively) were found in the time course. Conversely, a greater improvement in Rotigotine + MIRT group was observed for 6MWT (*p* < 0.0001) and TUG (*p* = 0.03). Along time, the dosage of Rotigotine was higher in patients who did not undergo MIRT, at all observation times following T0.

**Conclusions:**

Over the course of 18 months, the effectiveness of the combined treatment (Rotigotine + MIRT) on the patients’ global clinical status, evaluated with total UPDRS, was not inferior to that of the pharmacological treatment with Rotigotine alone. Importantly, rehabilitation allowed patients to gain better motor performances with lower DRT dosage.

## Introduction

Parkinson’s disease (PD) is one of the most common progressive neurodegenerative diseases. It is classically defined as a disorder related to degeneration of dopamine producing neurons in the pars compacta of the substantia nigra. PD is typically characterized by dopamine-related motor disturbances such as tremor, rigidity and bradykinesia. The cornerstone of symptomatic treatment for PD is the dopamine replacement therapy (DRT), which works by restoring the physiological synaptic plasticity in the dopamine-denervated striatum [1]. l-dopa still remains the most effective drug for PD [2], but other dopaminergic agents, such as dopamine agonists (DAs), monoamine oxidase (MAO)-B inhibitors and catechol-*O*-methyl transferase (COMT) inhibitors are used in the clinical practice.

A relevant issue regarding DRT concerns its long-term use, as it might cause aberrant structural changes in the striatum leading to the development of motor and behavioural side effects (such as motor fluctuations, wearing-off, dyskinesias, dopamine dysregulation syndrome) [3], which worsen the patients’ clinical conditions and the quality of their lives [4]. The total amount of DRT represents another aspect worthy of consideration: Warren Olanow et al. [5] described a relative l-dopa threshold effect, and they found that the risk of developing dyskinesias and wearing-off was increased at l-dopa doses ≥ 400 mg/day.

In order to optimize the pharmacological management of PD over time, the DAs are often prescribed to “de-novo” patients [6]. Among all DAs, Rotigotine is the most recent and offers several potential advantages, including a rapid onset of action, a constant drug delivery and ease of use [7, 8].

While in early PD the predominant motor symptoms are dopamine-related, in the more advanced disease stages the involvement of other neurotransmitter systems [9] leads to the development of no dopamine-related disturbances, which are unresponsive to DRT [9].

Recently, several studies have shown the effectiveness of rehabilitation for PD [10–16], mainly on drug-resistant axial disturbances (such as postural and gait dysfunctions) [17–19]. Specific physical trainings may influence positively the neuroplasticity in the basal ganglia by modulating the cortico-striatal excitability, even in early PD stages [20–23]. In a previous study [14] we have demonstrated that a tailored, intensive, multidisciplinary and goal-based rehabilitation treatment (MIRT) slowed down the progression of motor symptoms and reduced the need for increasing DRT in a group of PD patients in early stage of disease.

Such kind of evidences confirms the relevance and the beneficial role of rehabilitation even in early PD.

We argued that the good synergism between an optimal DRT use and the rehabilitation efforts could be crucial for maximizing their positive effects on cortico-striatal plasticity and for reducing the occurrence of drug-related motor and behavioural side effects [14, 24].

In order to investigate and better define the potential advantages coming from the synergism between DRT and rehabilitation in treating PD, in this study we have investigated the short- and the long-term (18 months) effectiveness of MIRT in a group of “de-novo” patients treated with rotigotine as monotherapy, in comparison with a control group of patients treated with the same DA, but who did not undergo rehabilitation.

## Materials and methods

**Fig. 1:**
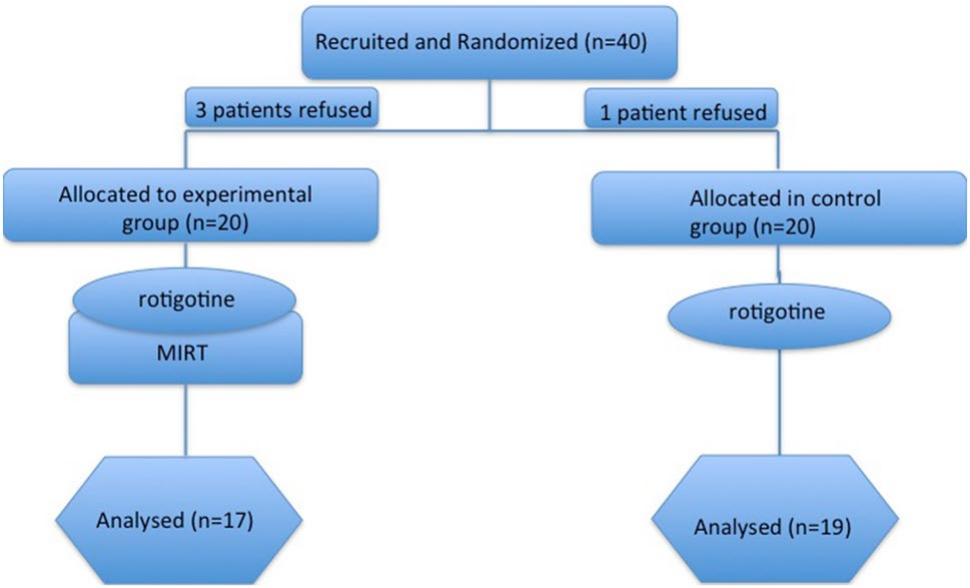
Experimental design

This was a multicenter, single blinded, parallel-group, 1:1 allocation ratio, randomized trial, aiming to compare the effectiveness of a treatment combining the use of the dopaminergic drug Rotigotine plus rehabilitation (Rotigotine + MIRT group) versus a pharmacological treatment alone, with the same DA (Rotigotine group) (see Fig. 1).

The hypothesis of this trial was that the synergistic association of the monotherapy with Rotigotine + MIRT, in a group of “de-novo” PD subjects, was, at least, as clinically effective as the Rotigotine alone, allowing at the same time a reduction in the need to increase the dopaminergic drug dosage along an 18-month observation period.

Hence, noninferiority of Rotigotine + MIRT with respect to Rotigotine alone was of interest on the premise that the first strategy might offer important advantages reducing the occurrence of drug-related motor and behavioural side effects. The study was, therefore, designed as a non-inferiority trial.

### Participants

The present study was conducted at the Department of Parkinson’s Disease, Movement Disorders and Brain Injury Rehabilitation of the “Moriggia-Pelascini” Hospital (Gravedona ed Uniti, Italy) and at the Parkinson’s disease Rehabilitation Center, “Fondazione Borghi” (Brebbia, Italy).

Forty “de-novo” patients with a diagnosis of idiopathic PD were considered eligible from expert neurologists in movement disorders. 36 of these accepted to participate and concluded the study (17 in the experimental group and 19 in the control group). Eligibility criteria were the following: (i) diagnosis of idiopathic PD according to the UK Brain Bank criteria [25], (ii) Hoehn and Yahr stage 1–2. Exclusion criteria were: (i) rotigotine intolerance and/or need to change the rotigotine monotherapy with other dopaminergic medications during the study period, (ii) any focal brain lesion detected with brain imaging studies (CT or MRI), (iii) psychosis (evaluated with Neuropsychiatric Inventory), (iv) auditory, visual and/or vestibular dysfunctions, (v) presence of comorbidities impairing the autonomy in activities of daily living.

The sample size was calculated assuming the same change in total UPDRS between the enrollment and the end of the study for both approaches, with a non-inferiority margin equal to 4.5 (i.e. the minimal clinically important difference [26] to achieve a power of 80%, with a one-sided type 1 error rate of 5%). This resulted in 32 patients (16 patients per group). Taking into account possible drop-outs, a sample size of 40 was deemed to be appropriate for this trial. Enrolled patients were randomly assigned to the Rotigotine group or to the Rotigotine + MIRT group using a computer-generated list of random numbers: 20 patients to the Rotigotine condition (Control Group) and 20 patients to the Rotigotine + MIRT condition (Experimental Group). The sequence was concealed until assignment, and the people enrolling participants did not know in advance which treatment the patient was assigned.

All eligible patients began treatment with Rotigotine, at the same dosage of 2 mg/24 h, which was then increased after 7 days to 4 mg/24 h. 4 weeks from the onset of Rotigotine treatment, following the randomization list, the control group continued to be treated only with the pharmacological treatment, while the other (Rotigotine + MIRT) underwent a 4-week MIRT. The same MIRT was repeated 1 year later.

**Fig. 2:**
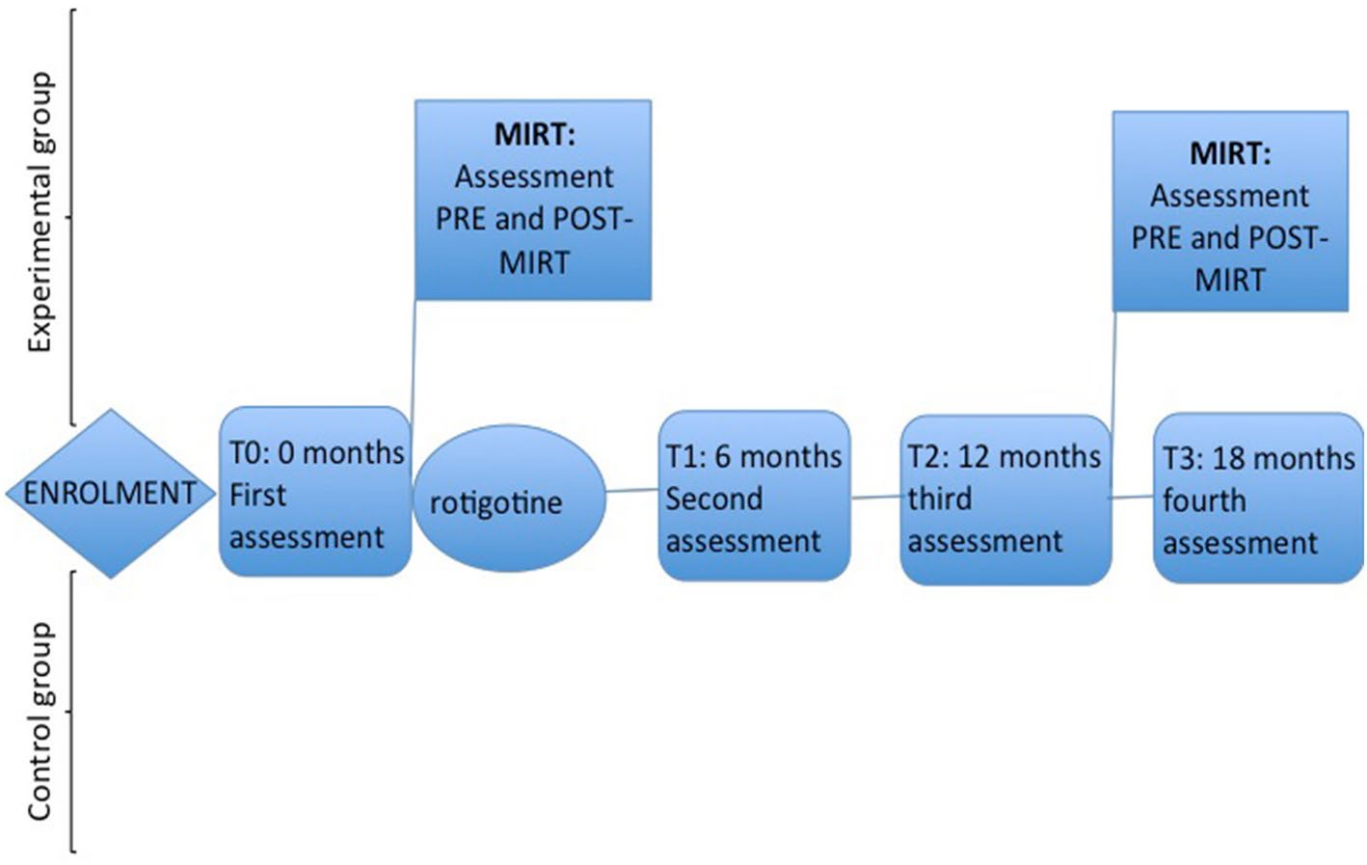
Study design

MIRT was performed at the Department of Parkinson’s Disease, Movement Disorders and Brain Injury Rehabilitation of the “Moriggia-Pelascini” Hospital (Gravedona ed Uniti, Italy). Patients enrolled in the control group did not undergo any rehabilitation treatment during the same study period (see Fig. 2). The study design and protocol were approved by the by the local institutional review board and by the Central Ethics Committee and were in accordance with the code of Ethics of the World Medical Association (Declaration of Helsinki, 1967). All patients signed an informed written consent prior to the participation to the study for the use of their clinical data for scientific purposes. This trial was registered on ClinicalTrials.gov website (NCT02100176).

### Outcome measures

Neurologists and physiotherapists expert in movement disorders, blinded to the treatment allocation and to the study design, assessed clinical, functional and motor scales. The primary outcome measure was the total score of Unified Parkinson’s Disease Rating Scale (UPDRS). The secondary outcomes included the UPDRS sub-sections II and III (UPDRS II–III), the Six-Minute Walk Test (6MWT), the Timed Up and Go Test (TUG) and the amount of DRT. All patients were evaluated at baseline (T0), 6 months (T1), 1 year (T2), and at 18 months (T3) (see Fig. 2). All evaluations were performed, in the morning, at about 9 AM, 1 h after the rotigotine transdermal patch placement. The assessment took part in the centre that previously enrolled the patient. During the 18-month study, the neurologist modified the pharmacological dosage on the basis of the patients’ clinical status and need. The dosage of rotigotine was recorded in both groups at the beginning of the study (T0), and after 6, 12, and at 18 months (T1, T2, T3, respectively).

Patients in the experimental group were also evaluated at the end of each 4-week MIRT.

### Rehabilitation treatment

MIRT is a multidisciplinary, aerobic, motor-cognitive, intensive and goal-based rehabilitation treatment specifically designed for PD patients [13, 14, 27]. The aim of the treatment was to re-learn the dysfunctional movements resulting from the disease through the use of explicit and implicit learning strategies. It consists of a 4-week program in a hospital setting, composed of four daily rehabilitative sessions for 5 days and 1 h of physical exercise on the sixth day. The duration of each session, including recovery periods, is about 1 h:

- The first session consists of a one-to-one treatment with a physical therapist. It comprises cardiovascular warm-up activities, active and passive exercises to improve the joints range of motion, stretching of the abdominal muscles, strengthening of paravertebral muscles, postural changes and exercises operating on balance and postural control.
- The second session exploits the use of various devices to improve gait and balance: a stabilometric platform with biofeedback (patients have to follow a pathway on a screen by using a cursor sensitive to their feet movements on the platform), a treadmill plus (treadmill training with visual cues, auditory cues and feedbacks) [28], a crossover [29] and a cycloergometer with feedback. We use a maximum treadmill speed of 3.5 Km/h; patients are trained with treadmill for no more than 15 min, two times per day.
- The third session consists of occupational therapy aimed to improve the autonomy in everyday activities. The session focuses on hand dexterity, writing and activities of daily living. The hand and finger dexterity training entails exercises aimed at re-acquiring the functional use of the most affected hand and the skills in the coordinated activities of both hands. The writing rehabilitation treatment consists of paper-and-pencil exercises and uses visual cues and verbal strategies aimed to enlarge the letters size and improve the readability. Finally, patients are trained by performing the activities of daily living in the rehabilitation setting, exploiting self-management and cognitive-behavioural strategies.
- The fourth session includes 1 h of speech therapy. In this field, three possible kinds of intervention are proposed: (i) a counselling for patients and caregivers pertinent to a good management of language and swallowing problems, (ii) an individual swallowing training, which includes meal monitoring and learning strategies for a correct ingestion of foods and liquids, (iii) a group therapy aimed to treat the hypokinetic dysarthria (breathing exercises to relax and alleviate the pressure of speech; facial exercises to improve the range of facial expressions and mouth motion; exercises to improve vocalization, articulation and speech prosody).

On the sixth day the patients are trained only with devices for 1.

The rehabilitation program could also include hydrotherapy in case of severe balance and postural disorders, robotic-assisted walking training for complex gait disorders, virtual-reality training and psychoeducational groups with neuropsychologists.

During all the activities, the heart rate reserve is kept between 70 and 80%.

A weekly team meeting defines the rehabilitation program for each patient and assesses its benefits during the course of the hospitalization.

### Statistical analysis

The Shapiro–Wilk statistic supported by visual inspection was used to test the normality of the distribution of all variables. No severe violations of the normality assumption were observed for the considered outcome variables. Descriptive statistics of collected data were reported as mean ± SD for continuous variables and as percentage frequency for categorical variables. Between-group comparisons for continuous variables were carried out by one-way analysis of variance. We examined differences in outcome measures using a two-factor repeated measures analysis of variance with treatment condition (Rotigotine and Rotigotine + MIRT) as the between factor and time of assessment (baseline—T0, 6-months—T1, 12-months—T2 and 18 months—T3 after enrolment) as the repeated, within factor. Post-hoc analyses were performed to compare follow-up and baseline measures within each group of patients and to compare between group outcomes at T3. The Tukey–Kramer adjustment for multiple comparisons was used. Adjusted p-values were reported when appropriate. In the Rotigotine + MIRT group the acute effect of MIRT was evaluated at the end of the first (PostMirt1) and second 4-week treatment (PostMirt2) by repeated measures ANOVA. Gender distribution was compared between groups using the Chi square test or Fisher exact test if appropriate.

The association between changes (T3––T0) in drug dosage and in functional variables was assessed by the Spearman correlation coefficient.

A 2-sided *p* value of 0.05 or less was considered statistically significant. All analyses were carried out using the SAS/STAT statistical package, release 9.4.

## Results

Figure 1 reports the flow chart of the progress through the phases of the study. Out of 40 eligible patients approached, 4 refused to participate in the study. There were no dropouts, leading to a final population of 36 patients, 17 in Rotigotine + MIRT group and 19 in the Rotigotine group. Since all patients completed the treatment according to the original allocation, intention to treat analysis and per protocol analysis coincide. Patients’ demographic characteristics are reported in Table 1. No differences in age and gender were observed across the two groups. Functional characteristics of studied patients at T0, T1, T2 and T3 observation times are reported in Table 2. The time between T2 and T0 (time distance between the two MIRT treatments in Rotigotine + MIRT group) was 12.3 ± 1.5 months.

**Table 1:** Demographic characteristics of studied patients

	All patients (*N* = 36)	Rotigotine + MIRT (*N* = 17)	Rotigotine (*N* = 19)	*p* value
Age (years)	64.5 ± 6.0	64.7 ± 7.3	64.4 ± 4.4	0.9
Male gender (%)	22 (61)	13 (76)	9 (47)	0.1

**Table 2:** Clinical, motor and functional characteristics of patients at four observation times: at baseline (T0), 6 months (T1), 1 year (T2), and 18 months (T3)

	Rotigotine + MIRT, T0	Rotigotine + MIRT, T1	Rotigotine + MIRT, T2	Rotigotine + MIRT, T3	Rotigotine, T0	Rotigotine, T1	Rotigotine, T2	Rotigotine, T3
Total UPDRS	30.2 ± 8.4	20.5 ± 11.1	27.8 ± 8.0	21.5 ± 5.3	30.0 ± 7.6	21.1 ± 7.5	27.7 ± 7.4	24.7 ± 6.0
UPDRS III	18.4 ± 5.9	12.8 ± 7.9	17.2 ± 5.1	12.5 ± 3.0	18.6 ± 4.9	13.2 ± 4.4	16.9 ± 4.8	13.4 ± 3.8
UPDRS II	8.1 ± 3.4	5.5 ± 3.6	8.5 ± 3.8	7.0 ± 2.4	8.4 ± 3.6	6.3 ± 2.6	8.8 ± 3.1	8.8 ± 2.4
6MWT (m)	361.2 ± 77.3	443.9 ± 89.3	414.9 ± 87.4	421.9 ± 80.7	344.7 ± 31.2	371.1 ± 32.7	372.1 ± 30.5	368.9 ± 32.1
TUG (s)	8.8 ± 1.7	7.5 ± 1.6	8.4 ± 2.1	8.1 ± 1.6	8.5 ± 1.2	8.1 ± 1.0	8.7 ± 0.9	8.4 ± 1.0
Rotigotine (dose in mg)	4.0 ± 0.0	4.6 ± 1.2	5.2 ± 1.6	5.4 ± 1.5	4.0 ± 0.0	5.6 ± 1.8	6.7 ± 1.4	7.2 ± 1.0

*UPDRS* Unified Parkinson’s Disease Rating Scale, *TUG* Timed Up and Go Test, *6MWT* Six-Minute Walk Test, *m* metres, *s* seconds

### Baseline

Baseline values of outcome measures were not different in the two groups (*p* = 0.95, *p* = 0.93, *p* = 0.83, *p* = 0.40 and *p* = 0.73 for total UPDRS, UPDRS III, UPDRS II, 6MWT and TUG, respectively).

Repeated measures ANOVA results for all variables are summarized in Table 3.

**Table 3:** F statistic and p-values for the main effects (Group and Time) and interaction from repeated measures ANOVA

	Group		Time		Group by time	
	F(1,34)	*p*	F(3,102)	*p*	F (3,102)	*p*
Total UPDRS	0.16	0.69	11.01	< 0.0001	0.83	0.48
UPDRS III	0.04	0.83	11.75	< 0.0001	0.19	0.90
UPDRS II	0.86	0.36	6.12	0.0007	0.99	0.40
6MWT (m)	5.77	0.022	4.83	0.003	8.39	< 0.0001
TUG (s)	0.66	0.42	2.54	0.06	2.95	0.03
l-dopa eq dose	11.19	0.002	44.11	< 0.0001	5.83	0.008

*UPDRS* Unified Parkinson’s Disease Rating Scale, *TUG* Timed Up and Go Test, *6MWT* Six-Minute Walk Test, *m* meters, *s* seconds

### UPDRS

Repeated measures ANOVA revealed that the group by time interaction was not significant for all UPDRS scores (total score, III and II, *p* = 0.48, *p* = 0.90 and *p* = 0.40, respectively), indicating no difference in the time course of these variables in the two groups. Indeed, total UPDRS scores in Rotigotine + MIRT group paralleled the scores in Rotigotine group and post hoc analysis revealed no significant differences at T3 in the two groups. Comparing the 95% confidence interval for total UPDRS at T3 with the pre-specified non-inferiority limit, non-inferiority was shown (*p* = 0.0001).

### 6MWT

A significant group by time interaction was observed for 6MWT (*p* < 0.0001) indicating that the time course of this variable over 1 year and a half period was different in patients who underwent Rotigotine + MIRT compared to those treated with Rotigotine alone. Post hoc analysis showed that the value of 6MWT was higher in patients who underwent MIRT at all observation times following T0. The difference between 6MWT values in Rotigotine + MIRT and Rotigotine at T1, T2 and T3 (unstandardized effect size) was 72.8 m (*p* = 0.0009), 42.8 m (*p* = 0.04) and 53.0 m (*p* = 0.01) at T1, T2 and T3, respectively.

### TUG

A significant group by time interaction was observed for TUG (*p* = 0.03). Post hoc analysis revealed that the only difference in TUG in the two groups was a higher improvement between T1 and T0 in the Rotigotine + MIRT group, but the difference between TUG values at T1 was only − 0.6 s and did not reach statistical significance (*p* = 0.16).

### Rotigotine

The time × treatment interaction was highly significant (*p* = 0.008). Post hoc analysis showed that the dosage of Rotigotine was higher in patients who did not undergo MIRT at all observation times following T0. The difference between Rotigotine dosage in Rotigotine + MIRT and Rotigotine group was − 1 mg (*p* = 0.02), − 1.5 mg (*p* = 0.0002) and − 1.8 mg (*p* < 0.0001) at T1, T2 and T3, respectively.

The total amount of Rotigotine was increased during the study in both groups, but at T3 in Rotigotine + MIRT group was increased by 30% as compared with T0, while in patients who did not undergo MIRT, the dosage was almost doubled, with an increase of 80%.

Considering the entire study period (T3 vs T0), we found in the Rotigotine group a correlation between DRT dosage and improvements in total UPDRS and UPDRS III (*r* = 0.48, *p* = 0.04 and *r* = 0.61, *p* = 0.005, respectively), but none with the motor performances evaluated with 6MWT (*p* = 0.19) and TUG (*p* = 0.45) scores. No correlation was observed for patients in the experimental group who gained better results in motor performances (*p* = 0.75, *p* = 0.83, *p* = 0.55 and *p* = 0.73 for UPDRS total, UPDRS III, 6MWT and TUG, respectively).

We also compared the effect of the two MIRT cycles in Rotigotine + MIRT group. Results are given in Table 4. Both MIRT were effective, and the improvement was significantly better in the first cycle for all variables except TUG.

**Table 4:** Changes in outcome measures in the experimental group after the first and the second MIRT

Variable	TPostMirt1-T0	*p* (TPostMirt1 vs T0)	TpostMirt2-T3	*p* (TpostMirt2 vs T3)	*p* (TpostMirt2-T3) vs (TPostMirt1-T0)
Total UPDRS	− 14.1 ± 5.6	< 0.0001	− 8.9 ± 4.9	<0.0001	0.0009
UPDRS II	− 3.9 ± 1.9	< 0.0001	− 2.6 ± 1.8	< 0.0001	0.040
UPDRS III	− 8.7 ± 4.3	< 0.0001	− 6.1 ± 5.1	0.0002	0.020
TUG (s)	− 1.7 ± 1.0	< 0.0001	− 1.1 ± 0.9	< 0.0001	0.14
6MWT (m)	81.8 ± 50.7	< 0.0001	48.1 ± 29.9	< 0.0001	0.038

TpostMIRT1vsT0: improvement after the first rehabilitation treatment; TpostMIRT2vsT3: improvement after the second rehabilitation treatment; TpostMIRT1-T3 vs TpostMIRT1-T1: differences between the improvement after the first and the second rehabilitation treatment

*UPDRS* Unified Parkinson’s Disease Rating Scale, *TUG* Timed Up and Go Test, *6MWT* Six-Minute Walk Test, *m* meters, *s* seconds, *TpostMIRT* Time Post Multidisciplinary Intensive Rehabilitation Treatment

## Discussion

This randomized, non-inferiority trial, aimed at comparing the short- and long-term (18 months) effectiveness of a treatment combining the use of the dopaminergic drug Rotigotine plus rehabilitation versus a pharmacological treatment alone with the same DA, in “de-novo” PD patients who have never been previously treated with any other dopaminergic drug.

### Main findings and novelties for the field

Five main findings raise from this study: (i) the effectiveness of the combined treatment (Rotigotine plus MIRT) on the patients’ global clinical status, evaluated with total UPDRS, was not inferior to that of the pharmacological treatment alone with Rotigotine; (ii) patients in the Rotigotine + MIRT group gained better performances at 6MWT and TUG; (iii) improvements in clinical performances were achieved with lower DA dose in comparison to the control group, so that patients who did undergo rehabilitation needed to increase dopaminergic drug dosage by 50% less than those treated only with the pharmacological therapy; (iv) considering the entire study period (T3 vs T0), we found in the Rotigotine group a direct correlation between DRT dosage and improvements in total UPDRS and UPDRS III, but we did not find a direct correlation with the motor performances evaluated with 6MWT and TUG scores. No correlation was observed for patients in the experimental group who gained better results in motor performances; (v) the improvement we found in gait performances (evaluated with 6MWT) was higher than the MDC (equal to 82 m, according to Steffen and Seney [30]), in 52.9% of subjects in the experimental group, while no one of those in the control group achieved this result, during the first study period.

As different preclinical or subclinical motor abnormalities have been found in early PD [31–33], these data appear to be even more relevant.

These results confirm the beneficial role of exercise for “de-novo” PD subjects: Christiansen et al. [34] have recently proposed that clinicians should consider the costs and benefits of exercise and activity behaviour interventions immediately after the diagnosis of PD. For these authors, this is essential to attenuate the health consequences of habitual low walking activity, that is typical in parkinsonian subjects compared with healthy people of similar age [35, 36]. Specifically, they found that step count in people with “de-novo” PD who were not regular exercisers approached sedentary lifestyle levels [34], thus suggesting that improving cardiorespiratory fitness might be an effective strategy to maintain healthy levels of walking activity and improve cardiorespiratory fitness [34]. In 16-month randomized controlled exercise intervention, Schenkman et al. [37] investigated three exercise approaches in individuals with early or mild stage PD: flexibility/balance/function exercise, supervised aerobic exercise, and home-based exercise. They showed overall functional benefits at 4 months in the flexibility/balance/function exercise group and improved walking economy (up to 16 months) in the aerobic exercise group [37]. It has been also observed that people with early PD participating in high-intensity body weight- supported treadmill training improve spatiotemporal gait parameters, kinematics of gait performance, and lower-extremity symmetry of ground reaction force in sit-to-stand task [23]. Consistently, it has been recently proposed that high-intensity treadmill exercise may be feasible and prescribed safely for patients with “de-novo” PD, but the authors conclude that an efficacy trial is warranted to determine whether high-intensity treadmill exercise produces meaningful clinical benefits in these patients [38, 39]. Beyond the different design and results, these studies were limited by the lack of a multidisciplinary, integrated motor-cognitive approach, which has been proposed to our patients and that is considered as central in the clinical management of PD and for patients’ quality of life [40–42]. It is also relevant that for this study we focused on individuals with “de-novo” PD and naïve to therapy, as it allows not only minimizing the confounding effects of medication on exercise intervention (as well as the confounding effect of other drugs different from Rotigotine for subjects in the Rotigotine group) but also the likelihood that patients would have functional limitations precluding exercise [39].

### Effectiveness of MIRT and Rotigotine: proposed mechanisms

Rehabilitation could be considered complementary to DRT and effective for the management of PD [10–16], especially for its positive effect on postural, balance and gait dysfunctions, well-known for their DRT-resistance [17–19]. Nevertheless, its essential role also for early PD subjects has already been widely described [14]. DRT partially improves the PD disturbances, mainly bradykinesia, tremor and rigidity [9]: these aspects represent only a part of the wide whole of disturbances that compose the PD spectrum [9], as the disease results in cognitive-motor abnormalities and in disorders of both movement expression and action performing. The main feature of the disease is the impaired ability to learn and express habitual-automatic actions [43–45]: since DRT does not improve the expression of habitual-automatic actions in Parkinsonian subjects, the main goal of rehabilitation should be properly the re-learning of the lost habitual motor behaviours. In fact, even though their habitual scheduling is altered [43], the parkinsonian subjects can still express habitual skills by using the executive-volitional component of action [28, 46–49]. For this reason, in the field of PD rehabilitation, external stimuli and specific techniques and strategies [28, 34–36, 50] have been properly developed in order to perform motor actions in a volitional and goal-directed manner [13, 14, 28, 49].

The improvements in motor performances following rehabilitation in PD could be related to activity-dependent neuroplastic changes [22, 51, 52]. Different variables, such as intensity, specificity, difficulty and complexity of exercise, have been recognized as fundamental for evoking this “activity-dependent neuroplasticity” in neurodegenerative disorders, including PD [53–55]. Nevertheless, the improvements in motor performances after MIRT cannot be explained only by these “activity-dependent” processes. The goal-directed approach of this kind of multidisciplinary and intensive rehabilitation is another fundamental requirement for achieve motor-functional improvements [49]. The physical techniques, the devices and the cognitive strategies adopted in this rehabilitative protocol, exploit the functions of the frontal cortical regions (specifically the pre-frontal cortex) and allow: (i) to bypass the dysfunctional basal ganglia, (ii) to stimulate the re-learning of the lost automatic movements, (iii) to reinforce the cortical mechanisms involved in the execution of the commands to move and, finally, (iv) to improve the patients’ mobility, balance, gait and posture [46, 49, 50, 56, 57].

Not least, it is conceivable that making exercise promotes proactive and resilient attitudes that could increase the patients’ sense of “self-management” of the disease.

With regard to Rotigotine, this DA not only offers advantages in term of onset of action, drug delivery and ease of use [7, 8], but it seems to have also a neuroprotective effect, as it has been demonstrated in the acute MPTP (1-methyl-4-phenyl-1,2,3,6-tetrahydropyridine) lesioned mouse model of PD [58]. Therefore, it is arguable that the use of Rotigotine possibly might maximize the benefits of motor exercises in terms of neuroprotection and neuroplasticity.

### The role of the synergism between DRT use and rehabilitative efforts

These results could be interpreted as a clear example of a good synergism between DRT use and rehabilitative efforts: the total amount of DRT at the end of the study was lower in patients who underwent rehabilitation, thus suggesting that the benefits obtained with MIRT reduce the need for increasing the dopaminergic drug dosage. Based on our results we hypothesize that MIRT plus DRT may delay or eliminate DRT side effects. Moreover, an optimal DRT titration is by itself fundamental for obtaining gains from rehabilitation: indeed, despite both DRT and different activity-dependent processes could restore the physiological synaptic plasticity in the dopamine-denervated striatum [1, 20, 54], the incautious and wrong DRT use could negatively influence the learning and reward processes, which are essential for rehabilitative purpose [59].

## Study limitations

There are some limitations of this study that have to be acknowledged. First of all, the design of this study could be considered as a limitation, due to the possible placebo effect. The placebo effect in PD is mediated by the release of dopamine in the dorsal striatum: the expectations of reward, such as those related to a novel pharmacological treatment or rehabilitation, seem to be particularly relevant for the “placebo-induced” clinical benefits [60, 61]. These benefits are strictly related to dopamine release in the ventral striatum, which leads to the activation of the reward circuitry [62]. The magnitude of the placebo-induced response likely depends on the “a priori” probability of clinical benefits [62]. This notion has profound implication in the design of clinical trials for PD [62]. Nevertheless, MIRT has been tested in previous studies and its effectiveness on motor, clinical and functional outcomes has been found to be closely related with objective metabolic measures [63] and with changes in the plasmatic levels of biochemical and molecular markers of neuroplasticity [22] so that the hypothesis of a placebo effect on outcome measures is unlikely.

Further, the study size is small, and this represents another limitation of the present study.

We did not use specific outcome measures for speech and dexterity despite the evidence that the intervention is a result of the total package, with presumed outcomes well beyond mobility. This should be considered as a study limitation. Nevertheless, among the secondary outcomes, we chose the total UPDRS, which allows evaluating the motor performance and different activities of daily life such as eating, dressing, writing, talking and the dexterity. Certainly, future studies with more appropriate outcome measures that more directly evaluate the effect of occupational therapy or speech therapy should be designed.

Finally, we did not perform any non-specific, non-intensive conventional rehabilitation in another group of PD patients in order to evaluate whether our results were related to the specific features of MIRT or could be interpreted as a general effects of exercise. Nevertheless, a number of literature data confirm how specific exercise features in terms of intensity and specificity are important to induce activity-related neuroplastic changes and clinical benefits [20–23, 51, 52, 54].

## Conclusion

We observed in a group of “de-novo” Parkinsonian subjects that, over the course of 18 months, the effectiveness of a combined treatment, Rotigotine plus MIRT, on the patients’ global clinical status was not inferior to that of the pharmacological treatment with Rotigotine alone. Nevertheless, over the time course, patients who underwent rehabilitation gained better performances at 6MWT and TUG and, above all, their need for increasing DRT was lower in comparison with patients treated only with the pharmacological therapy. These results underpin the importance of the synergism between DRT and rehabilitation in reducing the impact of the disease and the severity of symptoms, in controlling the short- and long-term DRT side effects and, finally, in promoting a pro-active attitude for the self management of the disease.
